# ^1^H, ^13^C, and ^15^N backbone chemical shift assignments of the C-terminal dimerization domain of SARS-CoV-2 nucleocapsid protein

**DOI:** 10.1007/s12104-020-09995-y

**Published:** 2020-12-03

**Authors:** Sophie M. Korn, Roderick Lambertz, Boris Fürtig, Martin Hengesbach, Frank Löhr, Christian Richter, Harald Schwalbe, Julia E. Weigand, Jens Wöhnert, Andreas Schlundt

**Affiliations:** 1 Institute for Molecular Biosciences, Johann Wolfgang Goethe-University Frankfurt, Max-von-Laue-Str. 9, 60438 Frankfurt/M, Germany; 2 Institute for Organic Chemistry and Chemical Biology, Johann Wolfgang Goethe-University Frankfurt, Max-von-Laue-Str. 7, 60438 Frankfurt/M, Germany; 3 Institute of Biophysical Chemistry, Johann Wolfgang Goethe-University Frankfurt, Max-von-Laue-Str. 9, 60438 Frankfurt/M, Germany; 4 Center for Biomolecular Magnetic Resonance (BMRZ), Johann Wolfgang Goethe-University Frankfurt, 60438 Frankfurt/M, Germany; 5 Department of Biology, Technical University of Darmstadt, Schnittspahnstr. 10, 64287 Darmstadt, Germany

**Keywords:** SARS-CoV-2, Structural protein, Nucleocapsid, Dimerization domain, Solution NMR-spectroscopy, Protein druggability, Covid19-NMR

## Abstract

The current outbreak of the highly infectious COVID-19 respiratory disease is caused by the novel coronavirus SARS-CoV-2 (Severe Acute Respiratory Syndrome Coronavirus 2). To fight the pandemic, the search for promising viral drug targets has become a cross-border common goal of the international biomedical research community. Within the international *Covid19-NMR* consortium, scientists support drug development against SARS-CoV-2 by providing publicly available NMR data on viral proteins and RNAs. The coronavirus nucleocapsid protein (N protein) is an RNA-binding protein involved in viral transcription and replication. Its primary function is the packaging of the viral RNA genome. The highly conserved architecture of the coronavirus N protein consists of an N-terminal RNA-binding domain (NTD), followed by an intrinsically disordered Serine/Arginine (SR)-rich linker and a C-terminal dimerization domain (CTD). Besides its involvement in oligomerization, the CTD of the N protein (N-CTD) is also able to bind to nucleic acids by itself, independent of the NTD. Here, we report the near-complete NMR backbone chemical shift assignments of the SARS-CoV-2 N-CTD to provide the basis for downstream applications, in particular site-resolved drug binding studies.

## Biological context

SARS-CoV-2 is the newest representative of the coronavirus family transmissible to humans and the cause of the COVID-19 respiratory disease. SARS-CoV-2, together with the closely related SARS-CoV (~ 79% sequence homology) and the Middle East Respiratory Syndrome (MERS)-CoV (~ 50% sequence homology), belongs to the genus of *Betacoronaviridae* and is one of seven known human-pathogenic CoVs (Chen et al. 2020). As of late September 2020, there have been more than 33 million confirmed cases of COVID-19 worldwide, causing more than 1,000,000 deaths according to the World Health Organization (https://covid19.who.int). These numbers strongly emphasize the urgent need for a vaccine as well as for potent viral inhibitors.

The positive-sense single-stranded RNA genome of SARS-CoV-2 is nearly 30 kb in length. It codes for 16 non-structural proteins (Nsp1 to 16) within the open reading frame (ORF) 1a/b, four structural proteins (Spike protein - S, Envelope protein - E, Membrane glycoprotein - M and Nucleocapsid - N) and several accessory proteins (Lu et al. 2020). Due to their role in the viral life cycle, the structural proteins as well as many of the central Nsps are highly promising targets for drug development. Besides the S protein, which is important for host cell entry, and the viral proteases Nsp5 (Mpro) and Nsp3d (PLpro), the N protein with its multiple crucial functions connecting transcription and RNA packaging represents a *bona fide* drug target.

The nucleocapsid protein consists of an N-terminal RNA-binding domain (NTD), preceded by a disordered part and followed by an SR-rich linker, and the C-terminal dimerization domain (CTD) followed by another disordered part. Its structural architecture is highly conserved among different coronaviruses. N-NTD and N-CTD are two independent domains that do not interact with each other (Chang et al. 2009), underlining their distinct functions. Both domains are able to bind nucleic acids (Chen et al. 2007; Huang et al. 2004; Zhou et al. 2020). In solution, the N protein mainly exists as a homodimer (Chang et al. 2005; Zhou et al. 2020), but based on the extended N-CTD it also tends to form less stable higher-oligomers (Ye et al. 2020), such as octamers, as was suggested for SARS-CoV (Chen et al. 2007). Upon self-association induced by the N-CTD, the N protein mediates viral RNA packaging (Luo et al. 2006). Furthermore, the involvement of the N-CTD in interactions with viral as well as host proteins has been demonstrated (Gordon et al. 2020; Kuo and Masters 2002).

Within the N protein oligomers, the dimeric CTD adopts a helical arrangement that could potentially mediate RNA condensation of the viral genome by presenting a continuous RNA-binding surface (Chen et al. 2007) along with stacked dimers. Indisputably, the dimer herein fulfills a crucial function, and its existence has been confirmed by multiple studies on SARS-CoV and SARS-CoV-2 including crystal structures and in vitro solution data (Chang et al. 2005; Luo et al. 2006; Chen et al. 2007; Takeda et al. 2008; Ye et al. 2020; Zhou et al. 2020). As of early October 2020, multiple crystal structures of the SARS-CoV-2 N-CTD have been described that all show an identical fold ((Ye et al. 2020; Zhou et al. 2020) and unpublished PDB IDs 6WJI, 6YUN, 6ZCO and 7CE0). The high degree of structural conservation of the CTD dimer is obvious from a structural comparison, e.g. of the SARS-CoV-2 PDB entry 7C22 (Zhou et al. 2020) with the SARS-CoV homolog represented by PDB entry 2CJR (Chen et al. 2007), yielding an RMSD of backbone atoms of 0.46 Å (not shown). Notably, an NMR structure (Takeda et al. 2008) of the latter protein directly confirmed its dimeric structure in solution. Consequently, the CTD appears as a valuable potential drug target, both by targeting the highly conserved dimer- and potentially higher-oligomer-interfaces and its binding sites for viral RNA during packaging. We here provide the near-complete backbone assignment of the SARS-CoV-2 N-CTD including the non-conserved SARS-CoV-2 specific residues. Our data are a valuable resource for atom-resolved solution analyses and the basis for residue-resolved screening applications.

## Methods and experiments

### Construct design

This study uses the SARS-CoV-2 NCBI reference genome entry NC_045512.2, identical to GenBank entry MN90894 (Wu et al. 2020). Domain boundaries for the N-CTD were defined in analogy to the available NMR structure (PDB 2JW8) of its closest homologue (96% identity), i.e. the N-CTD from SARS-CoV (Takeda et al. 2008). The expression construct used herein was designed to span amino acids 247–364 of the overall N protein primary sequence. An *E. coli* codon-optimized DNA construct coding for the SARS-CoV-2 N-CTD was obtained from Eurofins Genomics and sub-cloned into the pET3b-based vector pKM263, containing an N-terminal His_6_-tag and a GST-tag followed by a tobacco etch virus protease (TEV) cleavage site. After proteolytic TEV cleavage, the produced 13.6 kDa protein contained four artificial N-terminal residues (Gly-3, Ala-2, Met-1 and Gly0) preceding the start of the native protein sequence at Thr1 which corresponds to Thr247 in the full-length N protein sequence.

### Sample preparation

Uniformly ^13^C,^15^N-labelled N-CTD protein was expressed in *E. coli* strain BL21 (DE3) in M9 minimal medium containing 1 g/L ^15^NH_4_Cl (Cambridge Isotope Laboratories), 2 g/L ^13^C_6_-D-glucose (Eurisotop) and 100 μg/mL ampicillin. Protein expression was induced at an OD_600_ of 0.8 with 1 mM IPTG for 18 h at room temperature. Cell pellets were resuspended in 50 mM sodium phosphate, pH 7.4, 150 mM sodium chloride, 10 mM imidazole, and 100 µL protease inhibitor mix (SERVA) per 1 L of culture. Cells were disrupted by sonication. The supernatant was cleared by centrifugation (30 min, 9000 × g, 4 °C). The cleared supernatant was passed over a Ni^2+^-NTA gravity flow column (Sigma-Aldrich) and the His_6_-GST-tag was cleaved over night at 4 °C with 0.5 mg of TEV protease per 1 L of culture, while dialyzing into fresh buffer (50 mM sodium phosphate, pH 7.4, 150 mM sodium chloride, 10 mM imidazole). TEV protease and the cleaved tag were removed via a second Ni^2+^-NTA gravity flow column, and the N-CTD was further purified via size exclusion on a HiLoad 16/600 SD 75 (GE Healthcare) in size exclusion buffer (25 mM sodium phosphate, 50 mM sodium chloride, 0.5 mM EDTA, 0.02% NaN_3_, pH 6). Pure N-CTD protein containing fractions were determined by SDS-PAGE. According to its retention volume in the size exclusion chromatography the 13.6 kDa-protein is a dimer in solution. This is in line with recent publications on the SARS-CoV-2 N-CTD [in particular see (Zhou et al. 2020)]. Based on calibration, the peak position of the CTD corresponded to an approximate size of 26 kDa, which is in good agreement with the theoretical molecular mass of the dimeric protein (26.8 kDa). The SEC fractions of interest were pooled and concentrated using Amicon® centrifugal concentrators (molecular weight cutoff 10 kDa). NMR samples were prepared in 25 mM sodium phosphate, 50 mM sodium chloride, 0.5 mM EDTA, 0.02% NaN_3_, pH 6, 5% (v/v) D_2_O and 100-300 μM 4,4-dimethyl-4-silapentane-1-sulfonic acid (DSS) as internal chemical shift standard at N-CTD concentrations of 0.45 mM.

### NMR experiments

Backbone and Trp side chain amide assignments were performed by analyzing ^1^H,^15^N-HSQC and ^1^H,^15^N-TROSY experiments, the triple-resonance HNCACB experiment, and verified by the HN(CA)CO/HNCO pair of spectra (Clubb et al. 1992; Schleucher et al. 1993). For the HNCACB, the semi-constant-time (^15^N) triple-resonance pulse sequence applied in this study was ^1^H,^15^N-TROSY-based (Pervushin et al. 1997; Salzmann et al. 1998) and used sensitivity-enhanced gradient echo/antiecho coherence selection (Czisch and Boelens 1998; Schleucher et al. 1994; Schulte-Herbruggen and Sorensen 2000). Acceleration of longitudinal ^1^H relaxation between scans was achieved in the Band-Selective Excitation Short-Transient (BEST) (Lescop et al. 2007; Schanda et al. 2006) manner using exclusively shaped proton pulses with bandwidths/offsets of 5.0/8.4 ppm, respectively. The inter-scan delay was set to 0.3 s.

A ^15^N-NOESY-HSQC (Marion et al. 1989; Zuiderweg and Fesik 1989) with water suppression using a WATERGATE sequence (Piotto et al. 1992) was recorded to complete the assignment. The {^1^H}-^15^N heteronuclear NOE experiment was performed as an interleaved pseudo-3D TROSY version (Lakomek et al. 2012) using 128 complex points in the indirect dimension. All NMR experiments were carried out at 303 K with Bruker Avance III spectrometers of 600 and 950 MHz proton Larmor frequency, equipped with cryogenic probes and using Z-axis pulsed field gradients. Data acquisition and processing was undertaken using Topspin versions 3 and 4. Cosine-squared window functions were applied for apodization in all dimensions. Spectra were referenced with respect to internal DSS and for ^13^C and ^15^N as suggested in (Wishart et al. 1995).

### Assignments and data deposition

**Fig. 1:**
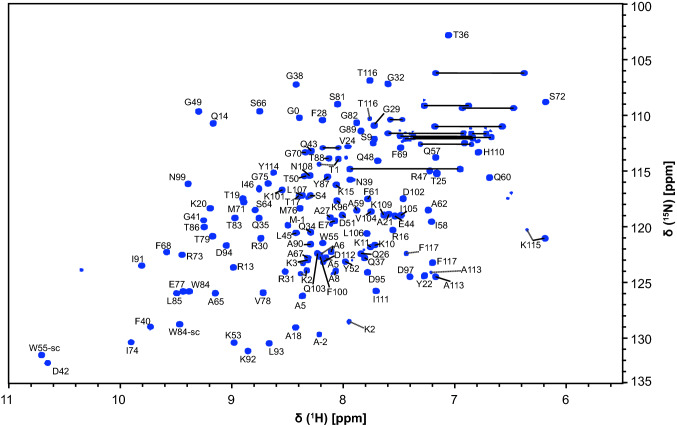
^1^H,^15^N-HSQC spectrum of the ^13^C,^15^N-labelled SARS-CoV-2 nucleocapsid C-terminal dimerization domain at 0.45 mM concentration in 25 mM sodium phosphate pH 6, 50 mM sodium chloride, 0.5 mM EDTA, 0.02% NaN_3_, 5% (v/v) D_2_O and 0.3 mM DSS collected at 303 K on a 950 MHz Bruker Avance III spectrometer equipped with a triple-resonance TCI cryogenic probe. Backbone NH peaks are labelled with their assignments. Trp side chain amides are indicated by W-sc. Straight lines indicate side chain amide pairs

Assignments of the dimeric N-CTD were performed using the CCPNMR analysis 2.4 software suite (Vranken et al. 2005) and the program Sparky (Lee et al. 2015). The ^1^H,^15^N-HSQC of N-CTD shown in Fig. 1 shows well dispersed peaks, suggesting the N-CTD to be a homogeneously folded species. For convenience, residues were numbered starting with 1 corresponding to Thr247 in the natural sequence. The overall high quality of all spectra allowed the backbone assignment of > 98% of all residues within the natural sequence (Thr1-Pro118, corresponding to Thr247-Pro364), and all Trp sidechain amides. Based on the high sequence similarity (Yoshimoto 2020), the assignments allow comparison to those of the previously published assignments for the 2002 SARS-CoV N-CTD_248-365_ (Takeda et al. 2008), showing broad agreements in amide chemical shifts. For the SARS-CoV-2 N-CTD we find a second, minor population for the backbone amides of residues Thr1-Ala6 and the very C-terminal stretch between Ala113 and Phe117 (Fig. 1). For the latter, we assume that this occurrence is due to the *cis*/*trans* equilibrium based on Pro118. For the N-terminal stretch, we hypothesize a second conformation in line with the merely tentative helical nature of this oligopeptide.

We could not assign the amide groups of His54 and Asn23. Notably, the amide group of His54 was also non-assignable in the analogous SARS-CoV N-CTD, while for Asn23 authors were successful in assigning the amide, but no additional atoms during the process of structure determination [BMRB entry 15511 (Takeda et al. 2008)], indicating line-broadening, likely based on chemical exchange within these solvent-exposed residues. Importantly, all of the five residues differing between SARS-CoV and SARS-CoV-2 N-CTD could unambiguously be assigned by us in the present study.

**Fig. 2:**
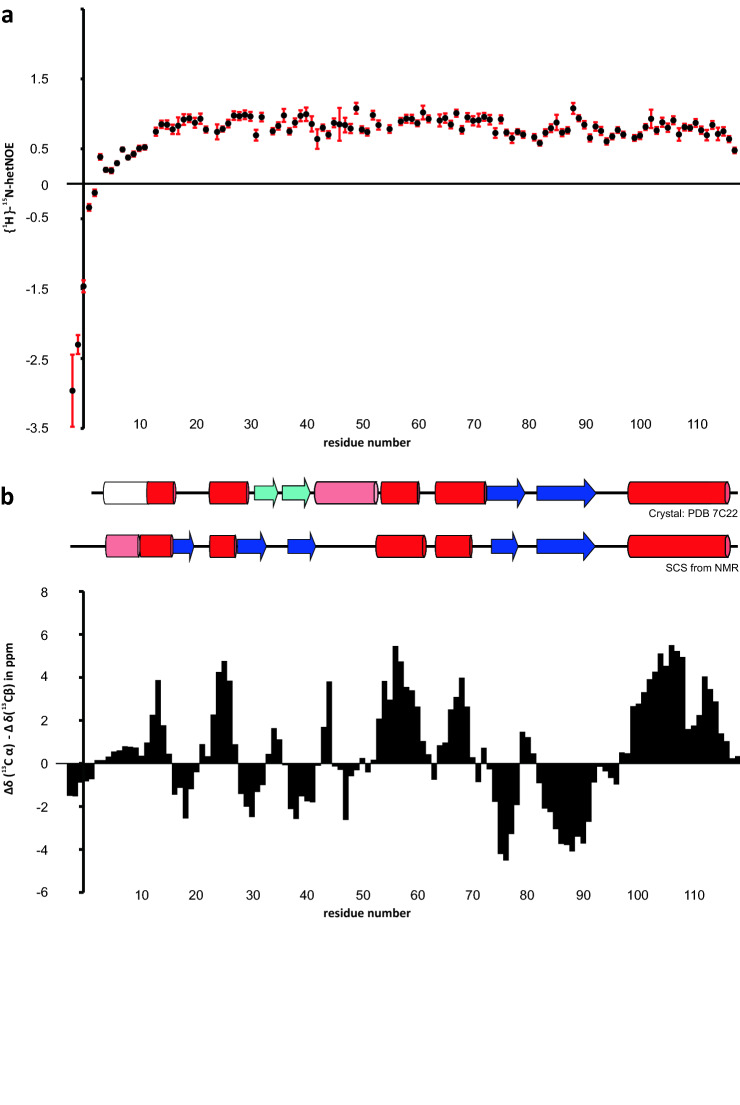
Display of {^1^H}^15^N heteronuclear NOE values (**a**) and combined Cα/Cβ carbon secondary chemical shift (SCS) values of the SARS-CoV-2 N-CTD plotted against the protein primary sequence as suggested by (Metzler et al. 1993) (**b**). (**a**) hetNOE values are shown with errors as derived from the program CCPNMR Analysis 2.4 (Vranken et al. 2005). No values are shown for the non-assigned residues Asn23 and His54. Additional gaps derive from prolines. (**b**) SCS are interpreted towards their underlying secondary structure as shown above the panel (experimental) and when compared to the SARS-CoV-2 N-CTD structure from PDB entry 7C22 (Zhou et al. 2020). α-helices are shown with red bars, β-strands with blue arrows, respectively. Light colors indicate the presence of elements with imperfect geometry in the structure or merely tentative secondary chemical shifts. Additional information on secondary structural elements within the N-terminal 11 residues, available from SARS-CoV-2 N-CTD structure from PDB entry 6YUN (unpublished), has been included as white bar.

To visualize internal dynamics within the N-CTD, we recorded hetNOE data as a function of the primary sequence (Fig. 2a). Comparable with the N-CTD of SARS-CoV, residues 1-11 display stepwise increasing rigidity in solution (Takeda et al. 2008), while only residues 13-118, which follow Pro12, show hetNOE values of 0.65 or higher; with the exception of Gly82, Asp94 and the very C-terminus beyond residue Lys115. Interestingly, the N-terminal amino acids 4-13 are present as an α-helix in some available crystal structures (here compared to PDB 7C22 (Zhou et al. 2020)), and our analysis of SARS-CoV-2 secondary chemical shifts (Fig. 2b) indicates this tendency also in solution (see below), while we only find a fully structured protein backbone starting from residue 13. Within the remainder of the protein, we find minor fluctuations of hetNOE values that are ascribable to loop regions, e.g. the stretch between residues 42 and 52 showing lower hetNOE values on average. Altogether, the SARS-CoV-2 N-CTD encompasses an overall rigid structure. No regions of increased flexibility were observed at the C-terminus of the construct, in line with the crystal structure of PDB entry 7C22. We also calculated carbon secondary chemical shifts based on the chemical shifts of C_α_ and C_β_ (Fig. 2b) relative to random coil values essentially as described by (Wishart and Sykes 1994). Four consecutive residues with significant negative (i.e. < − 1) or positive shifts (i.e. > 1) were used to define either β-strands or α-helices, respectively. Our data suggest a αβαββααββα-fold, which is in agreement with the representative crystal structure of PDB entry 7C22 (Zhou et al. 2020) (Fig. 2b). Notably, the last β-strand (residues 82 to 92) is involved in the formation of the hydrophobic dimeric interface, which is identical in the crystal and a SARS-CoV NMR structure (Takeda et al. 2008) of the N-CTD. Two additional differences between our NMR data and the SARS-CoV-2 crystal structure are remarkable though; we find a short β-strand (residues 16–20) extending from the initial stable helix (11–14), which is incompletely formed in the crystal structure. This stretch is part of a positively charged region, spanning the sequence from Thr1 to Pro33, that has been described to be involved in unspecific RNA interactions of the CTD (Zhou et al. 2020). Strikingly, we do not find an α-helix between residues 42 and 52, which is embedded in the above-mentioned RNA interaction site and thus distant from the dimer interface. This helix - albeit imperfectly - is, however, present in all crystal structures of SARS-CoV-2, and also in the NMR structure of SARS-CoV. Notably, in our SARS-CoV-2 NMR backbone data this stretch still unambiguously exhibits structure and compactness, but no secondary structure, indicating potential crystal artefacts. It remains to investigate the geometry by determining atom-resolved solution structures of SARS-CoV-2 N-CTD.

In summary, we find minor deviations from the crystal structure regarding the precise positioning of secondary structure elements in the amino-terminal half of the CTD, while the location of elements beyond residue 55 is identical in solution and the crystal state. Our NMR resonance assignments and the demonstrated spectral quality will now clearly pave the way towards a solution structure, RNA and protein interaction studies, and residue-resolved high-throughput drug screening as a crucial contribution to the initiative of screening all SARS-CoV-2 proteins as drug targets.

The chemical shift values for the ^1^H, ^13^C and ^15^N resonances of SARS-CoV-2 N-CTD have been deposited at the BioMagResBank (https://www.bmrb.wisc.edu) under accession number 50518 and are also accessible through https://covid19-nmr.de.
